# Outcomes of Treatment With Long-Acting Buprenorphine Injection in Individuals With Opioid Use Disorder Attending a Rehabilitation Center in the UAE

**DOI:** 10.1192/bjo.2024.471

**Published:** 2024-08-01

**Authors:** Hossameldin Tolba, Wael Foad, Abdulrazaq Ameri, Saeed Abdullah, Samer El Hayek

**Affiliations:** 1 Erada Center for Treatment and Rehabilitation, Dubai, UAE; 2 Dubai Medical College for Girls, Dubai, UAE; 3 Department of Psychiatry, American University of Beirut Medical Center, Beirut, Lebanon

## Abstract

**Aims:**

Opioid use disorder (OUD) is a global burden with significant morbidity and mortality. Standard of care often includes integrated treatment programs combining psychosocial interventions and Medication Assisted Therapy (MAT) which includes methadone, Buprenorphine (BUP) and Naltrexone. BUP, a partial u-opioid receptor agonist, has shown to increase patient treatment retention, reduce relapse, and improve quality of life. BUP Oral formulations can be associated with misuse, diversion, and non-adherence. Despite availability, many individuals don't receive adequate MAT treatment or discontinue medications prematurely, substantially increasing their relapse risk. Subcutaneous Long-Acting BUP (SC LABUP) injectable formulations have been associated with improved access, less burden of adherence, and greater abstinence in OUD patients. From this perspective, the OUD program at Erada Center maintains affected individuals on weekly or monthly SC LABUP injections. Our study aims to evaluate abstinence and treatment retention in Erada Center patients who are maintained on LABUP injections.

**Methods:**

We conducted a retrospective cohort study of all individuals following at Erada Center from January 2023 until January 2024, who were maintained on weekly or monthly LABUP injection. 174 individuals were identified, with diagnosis of OUD as per ICD–10 criteria, and receiving LABUP injection during inpatient admission or outpatient follow up. Primary outcomes were abstinence period (defined as negative urine drugs test apart from q-BUP), and retention in treatment (defined as compliance with attendance with OUD program). These were assessed at three time intervals: 24, 36, and 48 weeks from taking the first LABUP injection.

**Results:**

174 individuals were maintained on LABUP injection. Participants were all males, aged 18–65 years old, and polysubstance users with opioids being their drug of choice.

70 patients completed at least 24 weeks and received at least 2 doses of LABUP. Out of those, 53 achieved full abstinence and retention in 24 weeks (75.71%), 32 patients achieved the same for 36 weeks (45.71%), 25 patients achieved the same through 48 weeks (35.71%). Reasons for being lost to follow-up included relapse, incarceration (military service or custodial sentence), or drop out for no identifiable reasons.

**Conclusion:**

To the best of our knowledge, this is the first study in the UAE and Arab world looking at the outcomes of individuals with OUD maintained on LABUP injection. Results highlight a notable abstinence and retention rates as above. Further studies should look at reasons for relapse and loss for follow-up.

